# GenoSee: a novel visualization tool for graphical genotypes

**DOI:** 10.1270/jsbbs.24041

**Published:** 2024-11-23

**Authors:** Shumpei Hashimoto

**Affiliations:** 1 Laboratory of Plant Breeding and Genetics, Graduate School of Agricultural and Life Sciences, The University of Tokyo, 1-1-1 Yayoi, Bunkyo-ku, Tokyo 113-8657, Japan

**Keywords:** graphical genotype, genotype data, data visualization, visualization tools

## Abstract

Visualizing genotypic data is essential in genetic research and breeding programs as it offers clear representations of genomic information, enhancing understanding of genetic architecture. This becomes especially critical with the emergence of next-generation sequencing (NGS) technologies, which generate vast datasets necessitating effective visualization tools. While traditional tools for graphical genotypes have been groundbreaking, they often lack flexibility and universal applicability. These tools encounter limitations such as user-customized visualization and compatibility issues across different operating systems. In this study, I introduce GenoSee, a novel visualization tool designed to address these shortcomings. GenoSee can handle phased and non-phased variant calling data, offering extensive customization to suit diverse research requirements. It operates seamlessly across multiple platforms, ensuring compatibility, and provides high-quality graphical genotypes. GenoSee facilitates deeper insights into genomic structures, thereby advancing genetic and genomic research, and breeding programs by enhancing accessibility to genetic data visualization.

## Introduction

A graphical genotype visually represents genetic information across chromosomes, either in their entirety or in specific regions, offering a clear and detailed depiction of the distribution of genetic variants along chromosomes. This visualization aids researchers in understanding genetic architecture and analyzing complex traits. It facilitates the identification of genetic variations, tracking inheritance patterns, and enables comparative studies across genotypes. Graphical genotyping finds particular utility in plant and animal breeding programs, genetic mapping, and evolutionary studies. The concept of using a graphical genotype to illustrate haplotype diversity among chromosomes gained widespread acceptance following its introduction by Young and Tanksley (1989) for mapping populations with restriction fragment length polymorphisms (RFLP) (Young and Tanksley 1989).

Tools such as GGT 2.0 (van Berloo 2008) and MapChart (Voorrips 2002) have been developed for creating graphical genotypes. While groundbreaking at their inception, these pioneering tools often lacked flexibility and customization options, offering only basic functionalities for genetic data visualization. They fell short of meeting the diverse and dynamic requirements of modern genetic research. Typically, these tools adopted a static, one-size-fits-all approach and were constrained by their reliance on specific operating systems, limiting their utility in varied research environments.

The advent of next-generation sequencing (NGS) technology has revolutionized genetics by enhancing the capacity to gather extensive genetic data. This technological leap underscores the critical need for efficient and effective visualization tools to comprehensively interpret complex datasets. Recent advancements in NGS have also improved phasing techniques, allowing genotype determination at the haplotype level (Browning and Browning 2011). This capability provides deeper insights into genetic structures and relationships, facilitating more detailed and accurate genetic analyses. Phasing has become integral to modern genetic research, enabling researchers to discern the sequences of alleles on a chromosome and their association with various traits. Despite advancements in NGS and phasing techniques, several existing graphical genotyping tools have failed to keep pace with these developments. This gap underscores the urgent need to update and enhance these tools to comprehensively support the latest genetic data analysis and visualization advancements. In this study, I introduce GenoSee, a novel visualization tool for graphical genotypes. This tool offers high-quality and publication-ready graphical genotypes, accommodating phased and non-phased variant calling data. GenoSee is an invaluable tool that exhibiting efficacy in a wide range of applications requiring graphical genotypes, spanning genetic research, comparative genomics, and genome breeding.

## Materials and Methods

### Implementation details

GenoSee was developed on an Apple MacBook Pro with an Intel Core i5 processor and 8 GB RAM. The software was written in Python 3 (Van Rossum and Drake 2009), requiring a minimum of Python version 3.6, along with the libraries Pandas (McKinney 2010), Numpy (Harris *et al.* 2020), Matplotlib (Hunter 2007) and tqdm (https://tqdm.github.io/). The latest version of GenoSee is available on the GitHub repository (https://github.com/hashimotoshumpei/GenoSee). The repository includes examples of command-line usage and detailed instructions on how to use the tool.

### Input file preparation

When visualizing variant calling data using GenoSee, the input file (comma separated value; CSV format) can be created from a standard variant calling format (VCF) file. Specifically, it can be converted using the “make_input_file_from_VCF.py” script located in the “tools” folder of the GitHub repository using the following command: “python make_input_file_from_VCF.py path/to/input/VCF path/to/output/CSV --add_marker_names”. The “--add_marker_names” flag enable the automatic assignment of marker names by concatenating the chromosome name and position with an underscore (*e.g.*, Chr01_123456).

### Pre-constructed database

GenoSee includes a default database of chromosome lengths for eight species: *Oryza sativa* L., *Sorghum bicolor* (L.) Moench, *Zea mays* L., *Triticum aestivum* L., *Hordeum vulgare* L., *Glycine max* (L.) Merr., *Solanum lycopersicum* L., and *Arabidopsis thaliana* (L.) Heynh. This database allows for the automatic generation of graphical genotypes for these species using the “--species” or “-s” option (Table 1). Chromosome lengths for each species were derived from genome FASTA files obtained from RAP-DB (Kawahara *et al.* 2013, Sakai *et al.* 2013) for *O. sativa* and Phytozome (Goodstein *et al.* 2012) for other species.

### Adding new species and color sets

To apply GenoSee to other species, the chromosome lengths of new species can be added to the database using genome FASTA files by running “add_chromosome_length_to _database.py” in the GitHub repository with the command line “python add_chromosome_length_to_database.py --fasta path/to/fasta/file --database path/to/chromosome_length_database.json --name new_species_name”. Additionally, by directly editing “color_set.json”, new color sets can be used for visualization. In this case, “GenoSee.py” should be run with the “--color_palette name_of_new_color_set” argument.

### Evaluation with large-scale real data

The published variant calling data of rice (*O. sativa* and *O. rufipogon* Griff.) (Wu *et al.* 2023), bread wheat (*T. aestivum*) (Vasudevan and Cloutier 2023), and sorghum (*S. bicolor*) (Miao *et al.* 2020) were used to evaluate GenoSee. VCF files were locally downloaded and used for visualization. For rice and sorghum, separated VCFs were first concatenated using the “vcf-concat” function in VCFtools. The three VCF files were filtered with VCFtools using the following settings: “--max-missing 0.7 --maf 0.05 --recode” (for bread wheat and sorghum), and “--max-missing 0.95 --maf 0.05 --recode” (for rice). After filtering, 3,093,566, 215,919, and 35,775 variants remained for rice, bread wheat and sorghum respectively, and were visualized via GenoSee. Regarding the filtered data for rice, the execution environment used in this study (described below) does not have enough memory size to visualize genotypes of all 74 accessions in “compare” mode. Therefore, downsampling was performed by randomly extracting 100,000 variants from the filtered data using the “shuf” command, and the resulting data were used for visualization. For the extraction of the subset data, which is composed of 5, 10, or 100 accessions, bcftools was used with the “view -S” option. The execution time and maximum memory size were calculated using the “/usr/bin/time” command for three times for each condition. These evaluations were performed using Python 3.12.2 with pandas 2.2.2, numpy 2.0.1 and tqdm 4.65.0 on the homebuilt PC (Ubuntu 22.04.4 LTS, Intel Core i5-12600K processor, 64GB RAM). The Python version was downgraded to 3.7 when running “make_input_file_from_VCF.py” utilizing pyenv, given the dependency of the pyVCF library on the Python version.

## Results

### Application overview

**Fig. 1.**
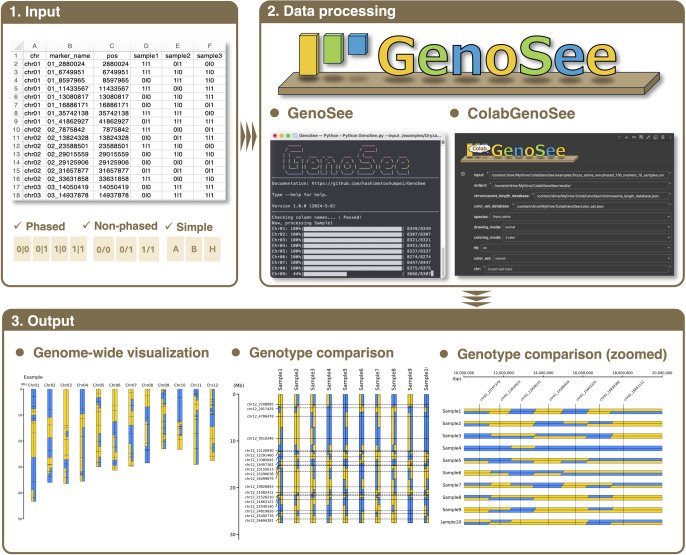
Overview of GenoSee. A simple CSV file is required as the data input. GenoSee accepts both phased and non-phased genotyping data. GenoSee utilizes the Python programming language and depends on several well-maintained Python packages during data processing. Visualization is facilitated using the Matplotlib library. GenoSee offers two user interface options: a command-line interface and a Jupyter Notebook via Google Colab (ColabGenoSee). In GenoSee, four visualization modes are supported: genome-wide visualization per sample (“normal” mode), comparison between samples (“compare” mode), and local comparison between samples (“zoomed” mode). These visualizations can be exported in PDF or PNG formats.

GenoSee is a high-quality visualization tool for graphical genotypes, designed to create user-friendly, customizable, and publication-ready graphical genotypes. It is available in two formats: a command-line version in Python (GenoSee) and a more interactive version in Google Colaboratory (Bisong 2019) (ColabGenoSee) (Fig. 1). Despite some limitations of Google Colaboratory, such as runtime duration (if using the free version), it offers features similar to web applications, including dropdown menus for selecting modes and colors (Fig. 1). All options available for visual selection in interactive mode can be specified using the arguments listed in Table 1 for command-line mode. The script has been intentionally simplified to allow easy customization, including changing visualization details, adapting to various plant species (see Materials and Methods), and changing color palettes using the Matplotlib library for high-quality figures. The input data for GenoSee should be in a CSV file, as shown in Fig. 1. The first four columns represent the chromosome number, marker name, and physical position, respectively. The fifth column and onward contain genotype information, with the column names representing arbitrary sample names (Fig. 1). Three genotypic formats are available for this tool: simple, phased, and non-phased. The simple genotype contains “A”, “B”, and “H”, representing two types (*e.g.*, two parental lines) of homozygous and heterozygous states, respectively. For both phased and non-phased genotypes, the representation of genotypes in the VCF file has been fully adhered to. For phased genotypes, the data should be in the following format: “1|1”, “0|0”, “1|0”, and “0|1”. For non-phased data, the genotypes are shown as “1/1”, “0/0”, “1/0”, and “0/1”. In these three formats, missing data are represented by “N”, “.|.”, and “./.”, respectively. The input data are processed using GenoSee, allowing graphical genotypes to be rendered according to the mode selected by the users, as shown in Fig. 1. In the current version of GenoSee (v1.0.0), polyploid genotypes (*e.g.*, 0/0/0/1) and multi-allelic genotypes (*e.g.*, 0/2) are not supported for visualization.

### Visualization results

**Fig. 2.**
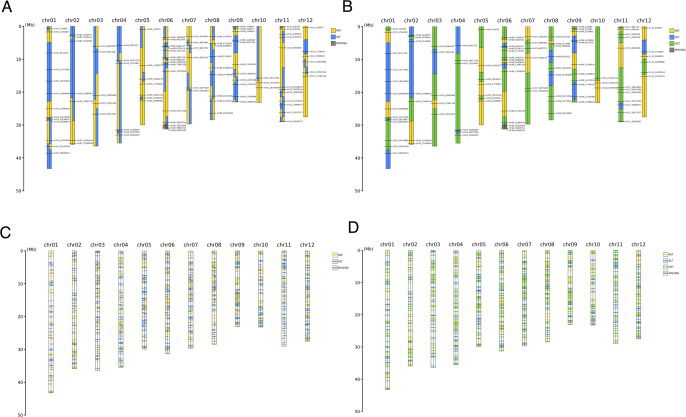
Representative visualization outputs from “normal” mode. If the “--fill” option is set to “on”, the space between each marker is filled with a suitable color that reflects the colors of the two adjacent markers (A, B). In contrast, when it is “off”, the genotype colors are displayed only at the marker positions, represented as horizontal lines (C, D).

**Fig. 3.**
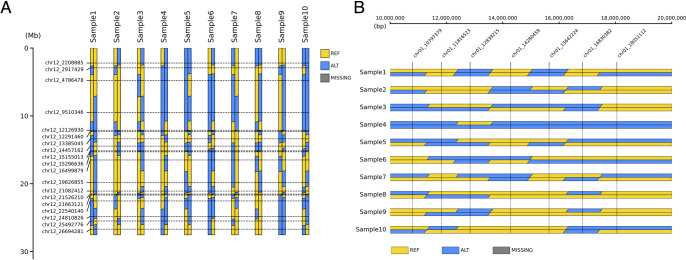
Representative visualization outputs from “compare” and “zoomed” modes. When the “compare” option is selected, each genotype of samples is aligned per each chromosome (A). “zoomed” mode enables the drawing of zoom-in graphical genotypes, which is helpful for local comparison of the genome or positional mapping of genes (B).

GenoSee can handle data from any species, regardless of chromosome count, or genome size, representing each chromosome as two narrow rectangles colored to indicate marker genotypes (Fig. 1). Even for species with polyploidy, as long as the genotype data is in a suitable format (see above), it can be used for visualization in GenoSee. This tool offers three types of graphical genotypes, specified by the “--drawing_mode” option. The first type (“normal”) is a genome-wide graphical genotype, useful for visualizing genotype variations across each chromosome (Fig. 2). This method facilitates material organization for genetic analyses and genomic breeding. The second type (“compare”) aligns chromosomes across multiple samples, enabling comparison of genotypes at the chromosomal level (Fig. 3A). Apart from its primary use, it serves as a visualization tool in comparative genomics. The third type (“zoomed”) offers a zoomed-in view of selected regions, facilitating genotype comparison between samples, particularly useful for genetic analysis, such as positional mapping (Fig. 3B). Two methods are available for coloring genotypes, selected using the “--coloring_mode” option. The first is the 2-color mode, displaying different genotypes in distinct colors (Figs. 2A, 2C, 3). When phased genotypes are used, phasing information is accurately depicted in the graphical genotype. The second is the 3-color mode, where heterozygous genotypes are represented by a third color (Fig. 2B, 2D). The “--fill” option determines whether areas between markers are filled (Fig. 2A, 2B). When turned off, only colored lines are drawn on each marker (Fig. 2C, 2D). As a rule of thumb, turning off the “--fill” option is advisable when the number of markers per chromosome exceeds 100.

### Capability to large-scale genotype data

**Fig. 4.**
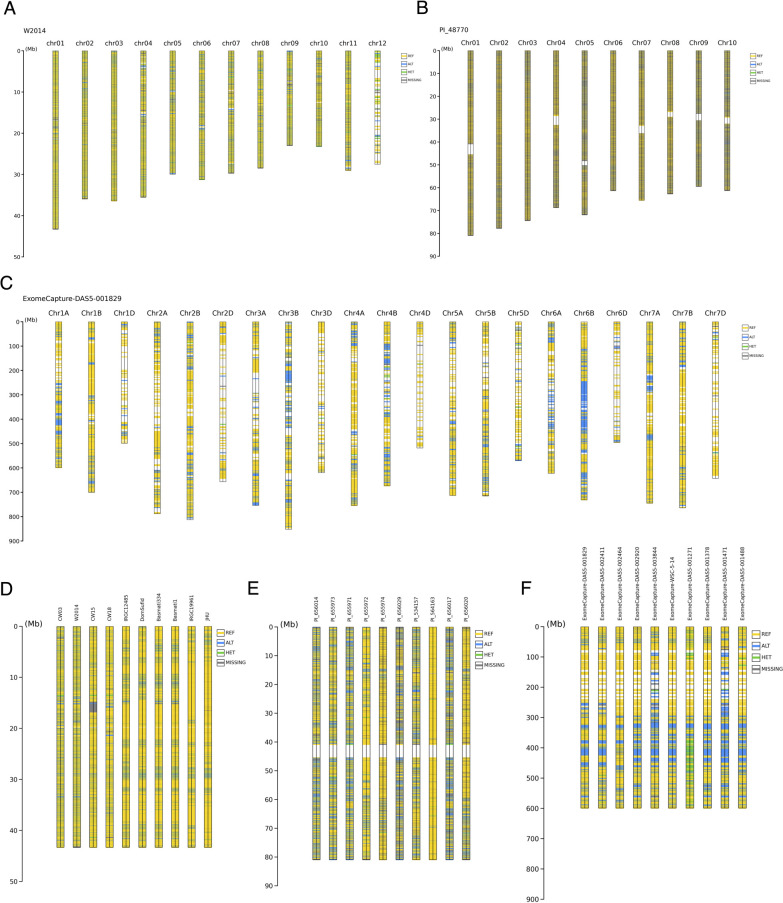
Visualization of large-scale genotype data. Variant calling data from rice (A and D), sorghum (B and E), and bread wheat (C and F) were visualized via GenoSee using the “normal” mode (A–C) and “compare” mode (D–F).

To verify the ability of GenoSee in handling large-scale real genotype data, an analysis and evaluation of variant calling data from three published datasets of rice (*Oryza sativa* and *O. rufipogon*) (Wu *et al.* 2023), bread wheat (*Triticum aestivum*) (Vasudevan and Cloutier 2023), and sorghum (*Sorghum bicolor*) (Miao *et al.* 2020) were conducted (Fig. 4, Table 2). The visualization results in both “normal” and “compare” modes for these datasets are presented in Fig. 4. The “normal” mode provided a comprehensive overview of the distribution and quantity of genome-wide variants at a glance (Fig. 4A–4C). In contrast, the “compare” mode facilitated a detailed comparison of variants between different accessions or cultivars across chromosomes (Fig. 4D–4F). This comparative visualization is crucial for identifying specific genetic differences and similarities across various samples. Furthermore, the execution time and maximum memory usage were evaluated during the visualization (Table 2). In the case of rice, visualizing data with 3,093,566 variants (approximately 250,000 variants per chromosome) required nearly two hours, even for just five samples. However, as demonstrated in the sorghum example, when the number of variants is limited to a few thousand per chromosome (in total, 35,775 variants), visualizations could be generated within half an hour even for 100 samples. These results demonstrate that GenoSee can visualize large-scale variant calling data. Because graphical genotyping is a method that primarily offers the advantage of visually comprehending the genomic structure overview, there is no need for an excessive number of variants. Therefore, to reduce the time required, when using variant calling data that include over ~100,000 variants per chromosome for visualization, it is recommended to either filter or randomly remove excessive variants in advance (see Materials and Methods).

### Visualization of introgression blocks using GenoSee

**Fig. 5.**
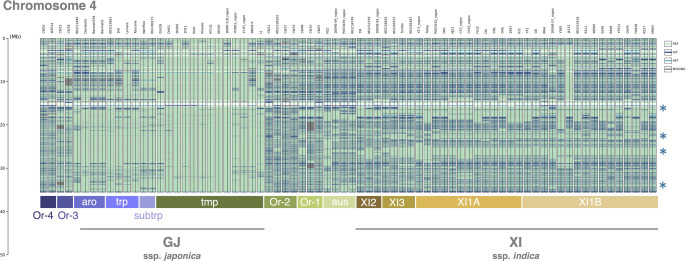
Introgression blocks (IBs) visualized via GenoSee. Variant calling data from Wu *et al.* (2023) was used for visualization. Asterisks indicate the positions of the large IBs (>300 kb) demonstrated in Wu *et al.* (2023). For visualization, “--color_set” option was set as “Aqua”.

To evaluate the potential applications of GenoSee in comparative genomics, the graphical genotype outputs generated in the previous section were analyzed. As previously mentioned, variant calling data obtained from Wu *et al.* (2023) was used as a validation data for rice. This genotype data includes 74 cultivated and wild rice accessions: *O. rufipogon* (n = 11), subspecies GJ (*O. sativa* ssp. *japonica*, n = 23), and subspecies XI (*O. sativa* ssp. *indica*, n = 40). Wu *et al.* (2023) identified genomic regions likely introgressed from ancient japonica to ancient indica or its wild ancestor, demonstrating that most domestication genes are present in these introgression blocks (IBs). For example, four large IBs (>300 kb) on chromosome 4 include domestications genes such as *LABA1* (Hua *et al.* 2015), *An-1* (Luo *et al.* 2013) (responsible for awn development), *sh4* (Li *et al.* 2006) (grain shattering), and *Bh4* (Zhu *et al.* 2011) (hull color). Therefore, whether GenoSee can visualize these IBs was further assessed. Notably, the major IBs reported in Wu *et al.* (2023) were clearly reproduced and visualized in the graphical genotype using GenoSee (Fig. 5).

## Discussion

Visualizing genomic data is crucial as it offers clear representations of genomic information, enhancing the understanding of complicated genomic architecture. The present study aimed to develop a novel visualization tool, GenoSee, to address the limitations of existing graphical genotyping tools and enhance visualization and analysis of genetic data. GenoSee is inherently flexible, supporting extensive customization options that empower researchers to modify visual outputs to align with their analytical needs. It is platform-independent, ensuring seamless utilization across different operating systems without compatibility issues. Tools similar to GenoSee, such as GGT 2.0 (van Berloo 2008) and MapChart (Voorrips 2002), have been previously reported. These packages have been used to create basic visualizations of genotypic data obtained for breeding selection or genetic studies. However, these programs have not been updated since 2007 and 2015, respectively, and have a low level of customization unsuitable for the current needs of genomics and genomic breeding, where variant calling data based on NGS technology are widely used. In particular, MapChart is limited to operating only on Windows OS, presenting a user-unfriendliness issue. As a relatively recent tool, Flapjack (Milne *et al.* 2010) is conceptually similar to my approach. It is routinely used to handle large volumes of data generated by high-throughput technologies, making it useful for interpreting large-scale data. However, it falls somewhat short in terms of the customizability of visualizations and figure quality and may not always be suitable for publication.

More recent tools, such as VIVA (Tollefson *et al.* 2019), VCF-Server (Jiang *et al.* 2019) and VCFshiny (Chen *et al.* 2023) can primarily analyze VCF files from NGS data. However, these tools lack the capability to render individual graphical genotypes and cannot visualize phased data. GenoSee overcomes these limitations by providing high-quality visualization in a user-friendly manner and supporting the utilization of variant calling data. The tool accommodates both phased and non-phased genotyping data, providing a detailed, customizable visualization of graphical genotypes. By bridging the gap between the rich data provided by NGS technologies and the need for detailed, adaptable visualization, GenoSee represents a significant advancement in genetic and genomic research.

In this study, as an example, I demonstrated that GenoSee is useful for visualizing introgression blocks. Using the data from Wu *et al.* (2023), I accurately reproduced the major introgression blocks, confirming the excellent ability of GenoSee in visualizing these genomic regions (Fig. 5). This study suggests the potential of GenoSee as a powerful tool not only for genetic analysis and genomic breeding in various species but also for elucidating the domestication and breeding history from genomic information. Therefore, GenoSee is the optimal tool for researchers aiming to efficiently and effectively generate graphical genotypes; GenoSee is all you need.

## Author Contribution Statement

SH conceived and designed the research, developed the software, conducted the analysis, and wrote the manuscript.

## Figures and Tables

**Table 1.** Options available in GenoSee v1.0.0, as shown by the command “Python GenoSee.py --help”

Flag	Description
-h, --help	Displays this help message and basic documentation.
-i, --input	Specifies the file path of the input CSV file (required).
-s, --species	Name of species to be analyzed (required).
-o, --output	Specifies the file path of the output image file.
--fill	Fill between markers (default: on).
--drawing_mode	Selects the drawing mode: normal, compare or zoomed (default: normal).
--coloring_mode	Selects the color mode: 2-color or 3-color (default: 2-color).
--color_set	Selects the color set (default: normal).
--display_marker_names	Adds marker names at each position (default: on).
--chr	Chromosome number of the region to zoom in.
--start	Start position of the region to zoom in.
--end	End position of the region to zoom in.
--dpi	dpi of the output image(s).
--pdf	Output as a PDF format (default: PNG only).

**Table 2.** Execution summary of visualization for different datasets using GenoSee

Organism	Ploidy	Genome size	Number of samples	Number of markers	Drawing mode	Execution time* (min.)	Max memory size* (GB)
*Oryza sativa*	2n = 2x = 24	~430 Mb	5	3,093,566	Normal	111.4	36.5
			5	3,093,566	Compare	111.4	35.0
			74	100,000	Compare	52.8	18.9
*Triticum aestivum*	2n = 6x = 42	~17 Gb	10	215,919	Normal	15.4	4.8
			10	215,919	Compare	15.4	4.2
			100	215,919	Compare	153.1	41.3
*Sorghum bicolor*	2n = 2x = 20	~730 Mb	10	35,775	Normal	2.7	0.9
			10	35,775	Compare	2.7	0.9
			100	35,775	Compare	26.0	7.9

* Average value of three measurements.
